# Influence of high-temperature exposure on the mating, oviposition and thermotaxis of *Bactrocera cucurbitae* (Coquillet) (Diptera:Tephritidae)

**DOI:** 10.1371/journal.pone.0204065

**Published:** 2018-09-20

**Authors:** Bei Zeng, Wenjing Zhu, Yueguan Fu, Shihao Zhou

**Affiliations:** 1 Institute of Tropical Agriculture and Forestry, Hainan University, Hainan, China; 2 Institute of Environment and Plant Protection, Chinese Academy of Tropical Agricultural Sciences, Hainan, China; Chinese Academy of Agricultural Sciences Institute of Plant Protection, CHINA

## Abstract

**Background:**

*Bactrocera cucurbitae* (Coquillett) is an important pest of cucurbit crops and certain vegetables in Asia, the Middle East, Africa and Hawaii. Most studies on *B*. *cucurbitae* have focussed on the effects of prolonged high temperature and very few have examined the effects of short-term exposures to high-temperature on behaviour.

**Results:**

In this study, short-term of high-temperature treatments of 33°C, 37°C, 41°C and 45°C were maintained for 1-3hr, and long-term, variable high-temperature treatments were established that consisted of experienced one, two and three times high temperatures stages to 31°C, 33°C, 34°C, 35°C, 36°C, 37°C, 41°C and 45°C for 7hr. We compared the effects of the different high temperatures regimes changes treatments on the mating, oviposition and thermotactic taxis of the flies. The results showed that exposure to a 45°C/1hr treatment, delayed both initiation of mating and oviposition for 8 hr relative to the control but mating and was observed 41 times and oviposition 47 times. By comparison, in the control, mating commenced immediately and was observed 38.3 times and oviposition was observed 41.3 times. Under the other treatments, all the indices for the flies declined with the increase in temperature and duration of exposure.

**Conclusion:**

Results showed that 1hr of exposure to 45°C significantly stimulated mating, oviposition and thermotactic behaviour of the flies. These results could improve our understanding of the mechanisms responsible for the population dynamics of *B*. *cucurbitae* during the high-temperature season.

## Introduction

Global warming has led to frequent occurrences of extremely high temperature in recent years. This may have both positive and negative impacts on feeding, mating, oviposition and thermotactic behaviour[1–3]. Insects encounter frequent short term episodes of high temperature. With changing climate, insect behaviour may change significantly. It is important to study the impact of short-term and long-term episodes of high temperatures[4–7].

Short-term or long-term changes high temperatures leads to hormesis of insects, namely, it is a survival tactic for insects adapting their behavior and physiology to cope with both short term and long term exposure to high temperatures[8–9].

Exposure to an extreme daily maximum temperature of 37–42°C in the summer changes the dietary behavioural rhythm of four ant species, including *Messor barbarous*, *Aphaenogaster senilis*, *Cataglyphis velox* and *C*. *rosenhaueri*, such that the behaviour shifts from foraging during the day and night to a type of bimodal twilight foraging that allows the ants to avoid the extreme high temperatures at noon[10]. An extreme daily maximum temperature also hinders the feeding behaviour of *Leucoptera coffeella*[11].

*Bactrocera cucurbitae* is a pest of economically important cucurbits and certain other vegetable crops[12]. It is distributed in tropical and subtropical zones of Asia, the Middle East, Africa and Hawaii. The pest populations usually explode during the high-temperature seasons[13–14]. *B*. *cucurbitae* adults tolerate 41–47°C but temperatures above 51°C are lethal. Adults are considered to be resistant to high temperatures[12]. In summer, the adult flies will oviposit before 10 am and then seek refuge under the leaves of the host plant at noon; after sunset, mating behavior commences and continues until the next morning. High-temperature conditions may influence the behaviour dynamics of this pest by inducing hormesis[12]. Most studies on *B*. *cucurbitae* have focussed on the effects of prolonged high temperature and very few have examined the effects of short-term exposures to high-temperature on behaviour[1]. In this paper, the effects on the mating, oviposition and thermotactic behaviour of this pest were studied in different high temperatures regimes, varied to replicate a range of field conditions.

## Materials and methods

### Insect rearing

*B*. *cucurbitae* were collected from a bitter gourd field in Chenmai County (110N; 19.75E), Hainan Province, China. No specific permissions were required for these locations/activities as those sites are not for a national park or other protected area of land, and also the field studies did not involve endangered or protected species. The flies were established in laboratory culture (i.e., 25°C; 14:10-h light: dark photoperiod; 60–80% relative humidity) to establish a stable experimental population. The larvae were fed on artificial diet (1L) fed with consisting of yeast powder (100g), corn powder(500g), sodium benzoate (2g), sucrose(100g), pumpkin(500g), concentrated hydrochloric acid (4 mL) and water (500mL). The adult of fruit flies artificial fed with yeast powder: sucrose (W:W) = 1:1. All experimental insects were newly emerged adults of this pest species.

### Reagents

Yeast powders, sodium benmzoate and hydrochloric acid were purchased from Shanghai Biology Engineering Technology Service Co., Ltd.

### Setting of short-term high temperature

The optimal temperature range for the growth, development, and reproduction of *B*. *cucurbitae* is reported to be 25–30°C[12]. In the experiments described, when the ambient temperature was >30°C, this was the lower value for the temperature range.

Adult *B*. *cucurbitae* were exposed to a range of temperatures in controlled temperature cabinets. The following different short-term high-temperature treatments were as follows: 33°C for 1hr (A_1_);33°C for 2hr (A_2_);33°C for 3hr (A_3_);37°C for 1hr (A_4_); 37°C for 2hr (A_5_); 37°C for 3hr (A_6_);41°C for 1hr (A_7_); 41°C for 2hr (A_8_); 41°C for 3hr (A_9_); 45°C for 1hr (A_10_);45°C for 2hr (A_11_) and 45°C for 3hr (A_12_); temperature treatments were conducted using a climate cabinet (Memmert HPP750 life Constant Climate Chamber) with 70±5% RH and a 14 L:10D photoperiod. The control condition (CK_1_) was set as 25°C for 1hr.

### Setting of the variable high temperature regimens

**Fig 1:**
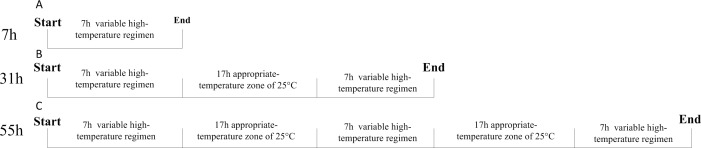
Setting of variable high temperature processing cycles.

To simulate temperatures in the field, the 45°C (recorded field maximum), 37°C (outdoor three-year average annual maximum temperature treatment) and 25°C (control) groups were used as the peak exposure temperatures. Flies in the first series of high temperature treatments (B_1-7_-B_6-7_) were exposed for a period of seven hours to a range of temperatures changing each hour to a high point then declining. The settings are listed in Table 1 (B_1_-B_6_). In the second and third series of exposure to high temperature regimens, flies were exposed to treatments similar to those in series 1 for two (B_1-37-_B_6-37_) and three (B_1-55_-B_6-55_) successive days respectively (Fig 1). Temperatures outside the seven hour periods of exposure to high temperature were maintained at 25°C to observe changes after the high-temperature treatment, we continued to observe each cage in an artificial climate cabinet. The 25°C for 7hr group was maintained as a control (CK_2_). This temperature regimen was repeated 3 times for each treatment.

**Table 1:** Setting of variable high temperature.

Scheme (°C, per hour)								Average value (°C)	
B_1_	31	33	37	**45**	37	33	31	35.286	model 1
B_2_	33	35	37	**37**	37	35	33	35.286	
B_1_	31	33	37	**45**	37	33	31	35.286	model 2
B_3_	33	37	41	**45**	41	37	33	38.143	
B_4_	31	33	35	**45**	35	33	31	34.714	
B_2_	33	35	37	**37**	37	35	33	35.286	model 3
B_5_	31	33	35	**37**	35	33	31	33.571	
B_6_	34	36	37	**37**	37	36	34	35.857	

Note: Model 1 is the same average temperature with different highest temperature treatments. Model 2 and model 3 are the same highest temperature with different average temperature treatments.

### Observations of the self-regulation of thermotactic behaviour of *B*. *cucurbitae* at high temperatures

As soon as the adult flies emerged, males and females were separated and 100 individuals were placed in homemade insect cages containing consistent water and an adult artificial diet. The flies in the cages were exposed to a range of short-term, high-temperature treatments and maintained at 70±5% RH with a 14L:10D cycle in an artificial climate cabinet. The artificial climate cabinet was designed with different surface temperatures in different parts of the cabinet. This allowed the flies to select their preferred resting place within the climate cabinet. We have delineated four temperature zone of 20–25° C, 25–30° C, 30–35° C and 35–40° C. At the end of the high temperature treatments, the flies resting in each temperature zone were recorded (individual thermotactic temperature zone trend ratio (%) = number of flies in a particular thermotactic zone /number of total individuals×100%). For example, if after 100 insects were exposed to 25°C temperature treatment, 50 flies choose to rest at a surface temperature zone of 20–25° C, this gives an individual thermotactic temperature zone trend ratio of 50%.

Experimental methods for observing the mating and oviposition behaviour of *B*. *cucurbitae* adult flies were arranged with 12 pairs of males and females placed in homemade cages containing water, an artificial diet and pumpkin slices for oviposition. The flies were placed in an artificial climate box at 70±5% RH and 14L:10D under the different short-term or long-term variable high-temperature conditions mentioned above. The time and frequency of the mating and oviposition behaviours within 9h following the high-temperature treatment were recorded. The mating of 12 flies constituted one replicate, and the treatment was repeated three times for each group.

### Statistical analysis

The inter-group comparison was performed using the SPSS data processing system software, completely randomized analysis of variance, and Tukey’s multiple comparisons.

## Results and analysis

### Effects of short-term high-temperature exposure on the thermotactic temperature behaviour of *B*. *cucurbitae*

The observations of thermotactic behaviour of the flies after the short-term, high-temperature treatments and CK_1_ were shown in Table 2 and Table 3. There was no significant difference in the temperature range values between females and males in the same treatments. The thermotactic temperature range values of the flies tended to decrease with longer observation times under the same temperature conditions. For the same observation time, temperature range values of the flies increase with increasing treatment temperatures when exposed for 1hr and 2hr. However, after 3hr of treatment, the temperatures were not significantly different, and the temperature trend range values were lower than those at 1hr and 2hr. These results were shown that the length of the exposure to high temperatures affects fly behaviour. The highest temperature range values were observed in flies exposed to the 45°C for 1h (A_10_) treatment with the rend temperature range of >40°C was 39% and 57% in males and females, respectively.

**Table 2:** Results of searching for favorite temperature behavior after short-term exposure to constant high temperatures.

Scheme			20–25°C(Number)	(%)	25–30°C(Number)	(%)	30–35°C(Number)	(%)	35–40°C(Number)	(%)	>40°C(Number)	(%)
CK_1_		♀	19	19.00%	81	81.00%	0	0.00%	0	0.00%	0	0.00%
		♂	32	32.00%	68	68.00%	0	0.00%	0	0.00%	0	0.00%
33°C	1hr(A_1_)	♀	0	0.00%	1	1.00%	99	99.00%	0	0.00%	0	0.00%
		♂	0	0.00%	2	2.00%	98	98.00%	0	0.00%	0	0.00%
	2hr(A_2_)	♀	0	0.00%	0	0.00%	100	100.00%	0	0.00%	0	0.00%
		♂	0	0.00%	2	2.00%	98	98.00%	0	0.00%	0	0.00%
	3hr(A_3_)	♀	0	0.00%	100	100.00%	0	0.00%	0	0.00%	0	0.00%
		♂	0	0.00%	100	100.00%	0	0.00%	0	0.00%	0	0.00%
37°C	1hr(A_4_)	♀	0	0.00%	1	1.00%	11	11.00%	88	88.00%	0	0.00%
		♂	0	0.00%	0	0.00%	21	21.00%	79	79.00%	0	0.00%
	2hr(A_5_)	♀	0	0.00%	0	0.00%	31	31.00%	69	69.00%	0	0.00%
		♂	0	0.00%	1	1.00%	24	24.00%	75	75.00%	0	0.00%
	3hr(A_6_)	♀	1	1.00%	99	99.00%	0	0.00%	0	0.00%	0	0.00%
		♂	0	0.00%	100	100.00%	0	0.00%	0	0.00%	0	0.00%
41°C	1hr(A_7_)	♀	0	0.00%	0	0.00%	5	5.00%	94	94.00%	1	1.00%
		♂	0	0.00%	0	0.00%	5	5.00%	95	95.00%	0	0.00%
	2hr(A_8_)	♀	0	0.00%	0	0.00%	7	7.00%	86	86.00%	7	7.00%
		♂	0	0.00%	0	0.00%	9	9.00%	91	91.00%	0	0.00%
	3hr(A_9_)	♀	1	1.00%	99	99.00%	0	0.00%	0	0.00%	0	0.00%
		♂	0	0.00%	100	100.00%	0	0.00%	0	0.00%	0	0.00%

Note:CK_1_ is the 25°C-1hr treatment.

**Table 3:** Results of searching for favorite temperature behavior after short-term exposure to constant high temperatures (continued).

Scheme			20–25°C(Number)	(%)	25–30°C(Number)	(%)	30–35°C(Number)	(%)	35–40°C(Number)	(%)	>40°C(Number)	(%)
CK_1_		♀	19	19.00%	81	81.00%	0	0.00%	0	0.00%	0	0.00%
		♂	32	32.00%	68	68.00%	0	0.00%	0	0.00%	0	0.00%
45°C	1hr(A_10_)	♀	0	0.00%	2	2.00%	3	3.00%	56	56.00%	**39**	**39.00%**
		♂	0	0.00%	3	3.00%	8	8.00%	32	32.00%	**57**	**57.00%**
	2hr(A_11_)	♀	0	0.00%	2	2.00%	16	16.00%	74	74.00%	8	8.00%
		♂	0	0.00%	2	2.00%	15	15.00%	79	79.00%	4	4.00%
	3hr(A_12_)	♀	1	1.00%	99	99.00%	0	0.00%	0	0.00%	0	0.00%
		♂	0	0.00%	100	100.00%	0	0.00%	0	0.00%	0	0.00%

### The effects of high temperature variation on the thermotactic behaviour of *B*. *cucurbitae*

The observations of thermotactic behaviour of the flies after the short-term, high-temperature treatments and CK_2_ were shown in Table 4 and Table 5. Among all the treatments, the thermotactic temperature range values had no differences between female and male adults. The temperature trend range is mostly concentrated at 25–30°C in all the treatments. For all treatments in B_4_, the range of trending temperatures is also centered on 20–25°C.

**Table 4:** Results of searching for favorite temperature zones behavior after exposure to varying high temperatures.

Scheme			20–25°C(Number)	(%)	25–30°C(Number)	(%)	30–35°C(Number)	(%)	35–40°C(Number)	(%)	>40°C(Number)	(%)
CK_2_		♀	19	19.00%	81	81.00%	0	0.00%	0	0.00%	0	0.00%
		♂	32	32.00%	68	68.00%	0	0.00%	0	0.00%	0	0.00%
B_1_	7hr	♀	0	0.00%	100	100.00%	0	0.00%	0	0.00%	0	0.00%
		♂	0	0.00%	100	100.00%	0	0.00%	0	0.00%	0	0.00%
	24hr	♀	0	0.00%	100	100.00%	0	0.00%	0	0.00%	0	0.00%
		♂	0	0.00%	100	100.00%	0	0.00%	0	0.00%	0	0.00%
	48hr	♀	0	0.00%	100	100.00%	0	0.00%	0	0.00%	0	0.00%
		♂	1	1.00%	99	99.00%	0	0.00%	0	0.00%	0	0.00%
B_2_	7hr	♀	0	0.00%	100	100.00%	0	0.00%	0	0.00%	0	0.00%
		♂	0	0.00%	100	100.00%	0	0.00%	0	0.00%	0	0.00%
	24hr	♀	0	0.00%	100	100.00%	0	0.00%	0	0.00%	0	0.00%
		♂	1	1.00%	99	99.00%	0	0.00%	0	0.00%	0	0.00%
	48hr	♀	1	1.00%	99	99.00%	0	0.00%	0	0.00%	0	0.00%
		♂	0	0.00%	100	100.00%	0	0.00%	0	0.00%	0	0.00%
B_3_	7hr	♀	0	0.00%	100	100.00%	0	0.00%	0	0.00%	0	0.00%
		♂	0	0.00%	100	100.00%	0	0.00%	0	0.00%	0	0.00%
	24hr	♀	1	1.00%	99	99.00%	0	0.00%	0	0.00%	0	0.00%
		♂	1	1.00%	99	99.00%	0	0.00%	0	0.00%	0	0.00%
	48hr	♀	1	1.00%	99	99.00%	0	0.00%	0	0.00%	0	0.00%
		♂	1	1.00%	99	99.00%	0	0.00%	0	0.00%	0	0.00%

Note:CK_2_ is the 25°C-7h treatment.

**Table 5:** Results of searching for favorite temperature behavior after exposure to varying high temperatures (continued).

Scheme			20–25°C(Number)	(%)	25–30°C(Number)	(%)	30–35°C(Number)	(%)	35–40°C(Number)	(%)	>40°C(Number)	(%)
CK_2_		♀	19	19.00%	81	81.00%	0	0.00%	0	0.00%	0	0.00%
		♂	32	32.00%	68	68.00%	0	0.00%	0	0.00%	0	0.00%
B_4_	7hr	♀	16	16.00%	84	84.00%	0	0.00%	0	0.00%	0	0.00%
		♂	16	16.00%	84	84.00%	0	0.00%	0	0.00%	0	0.00%
	24hr	♀	20	20.00%	80	80.00%	0	0.00%	0	0.00%	0	0.00%
		♂	23	23.00%	77	77.00%	0	0.00%	0	0.00%	0	0.00%
	48hr	♀	14	14.00%	86	86.00%	0	0.00%	0	0.00%	0	0.00%
		♂	15	15.00%	85	85.00%	0	0.00%	0	0.00%	0	0.00%
B_5_	7hr	♀	1	1.00%	99	99.00%	0	0.00%	0	0.00%	0	0.00%
		♂	0	0.00%	100	100.00%	0	0.00%	0	0.00%	0	0.00%
	24hr	♀	0	0.00%	100	100.00%	0	0.00%	0	0.00%	0	0.00%
		♂	0	0.00%	100	100.00%	0	0.00%	0	0.00%	0	0.00%
	48hr	♀	0	0.00%	100	100.00%	0	0.00%	0	0.00%	0	0.00%
		♂	0	0.00%	100	100.00%	0	0.00%	0	0.00%	0	0.00%
B_6_	7hr	♀	1	1.00%	99	99.00%	0	0.00%	0	0.00%	0	0.00%
		♂	0	0.00%	100	100.00%	0	0.00%	0	0.00%	0	0.00%
	24hr	♀	0	0.00%	100	100.00%	0	0.00%	0	0.00%	0	0.00%
		♂	1	1.00%	99	99.00%	0	0.00%	0	0.00%	0	0.00%
	48hr	♀	0	0.00%	100	100.00%	0	0.00%	0	0.00%	0	0.00%
		♂	1	1.00%	99	99.00%	0	0.00%	0	0.00%	0	0.00%

The results were shown that short-term exposure to a constant high temperature could improve the heat resistance of the flies compared to exposure to variable high temperatures and that the heat resistance of the flies was higher when exposed to different temperatures. The durations of the variable high-temperature and high-temperature treatments were too long to adversely affect the flies.

### Effects of short-term high-temperature exposure on mating and oviposition behaviour of *B*. *cucurbitae*

The observations of mating and oviposition behaviour of the flies after the short-term, high-temperature treatments and CK_1_ were shown in Table 6. After exposure to short-term high temperatures, the time before commencement of mating (N_1_) and oviposition (N_2_) were both delayed. Flies exposed to 45°C for 3hr (A_12_) showed the longest times before mating (528.3 mins) and before oviposition (518.7 mins), respectively. As the duration of high temperature exposure under the same temperatures increased, N_2_ and the frequency of mating (N_3_) were also affected. After 1hr, N_3_ and frequency of oviposition (N_4_) were highest after the 45°C exposure (A_10_), with up to 41 times and 47 times, respectively, for all the treatments. After the 2hr and 3hr treatments, the mating and oviposition frequencies were firstly increased and then decreased. After treatment at 37°C for 2hr (A_5_) and 37°C for 3hr (A_6_), N_3_ was increased by 34.3 times, and N_4_ was increased by 35.3 times.

**Table 6:** Results of time and frequency of mating and oviposition after short-term high temperatures.

Scheme	CK_1_	33°C			37°C			41°C			45°C		
		1h (A_1)_	2h (A_2_)	3h (A_3_)	1h (A_4_)	2h (A_5_)	3h (A_6_)	1h (A_7_)	2h (A_8_)	3h (A_9_)	1h (A_10_)	2h (A_11_)	3h (A_12_)
N_1_	4.7±0.3 i	268.2±3.7 h	287.3±1.5 g	323.5±3.8 e	297.1±1.1 fg	331.6±2.0 e	459.3±2.0 c	301.0±0.7 f	333.6±2.1 de	476.5±3.7 b	304.8±0.8 f	344.2±2.9 d	**528.3±1.7** **a**
N_2_	4.7±0.9 h	260.7±4.6 g	278.8±4.7 f	308.5±1.0 e	295.6±2.3 e	336.6±3.5 d	469.5±1.8 b	299.5±1.8 e	340.8±1.5 cd	482.5±3.8 b	302.4±1.2 e	350.9±2.3 c	**518.7±2.4** **a**
N_3_	38.3±0.3 ab	27.3±0.9 def	28.3±0.3 de	30.0±0.6 d	34.0±0.6 c	34.3±0.3 c	35.3±0.7 c	26.3±0.3 efg	26.7±0.3 efg	36.7±0.3 bc	**41.0±0.6** **a**	25.0±0.6 fg	24.0±0.6 g
N_4_	41.3±0.3 b	28.7±0.3 de	30.0±0.6 d	30.7±0.3 d	34.3±1.2 c	34.3±0.9 c	35.3±0.9 c	26.3±0.3 ef	28.3±0.3 de	39.0±0.6 b	**47.0±0.6** **a**	24.7±0.3 f	23.3±0.3 f

Note: Mating and oviposition behaviors were recorded within 9hr after different brief constant high temperature treatments and set time as N_1_, N_2_, the frequency as N_3_, N4. The data given is average ± standard deviation. Different small letters indicate a significant difference by Tukey’s new multiple range test at the P < 0.05 levels, respectively.

### Effects of high-temperature variation on mating and oviposition behaviour of *B*. *cucurbitae*

The observations of mating and oviposition behaviour of the flies after the short-term, high-temperature treatments and CK_2_ were shown in Table 7. The results are shown in Table 5. After different treatments of varying exposure to high temperatures, N_1_ and N_2_ were both delayed. With different durations of the same high temperature, N_1_ and N_2_ were delayed; under different high temperatures with the same treatment time, N_1_ and N_2_ increased first and then decreased. The results showed that for B_3-55_ (average temperature at 38.1°C) N_1_ and N_2_ were 487 mins and 457.9 mins respectively. N_3_ and N_4_ were highest after B_4-7_, increasing by 23.7 and 22.7 times, respectively. In general, high temperatures could affect the mating and oviposition behaviours of the flies. Varying high-temperature regimens were not conducive to the mating and oviposition behaviours of the flies when compared to the short-term high-temperature treatments. In this study, the mating and oviposition times of the flies significantly increased after exposure to temperatures of 45°C for 1hr, indicating that *B*. *cucurbitae* can improve its fecundity in response to temperature stress.

**Table 7:** Results of time and frequency of mating and oviposition after to exposure to varying high temperature.

Scheme	CK_2_	B_1-7_	B_1-31_	B_1-55_	B_2-7_	B_2-31_	B_2-55_	B_3-7_	B_3-31_	B_3-55_	B_4-7_	B_4-31_	B_4-55_	B_5-7_	B_5-31_	B_5-55_	B_6-7_	B_6-31_	B_6-55_
N_1_	12.5±1.3 j	407.6±1.1 g	434.6±2.3 d	471.3±0.4 b	395.8±0.9 h	426.5±2.0 de	468.2±0.5 b	418.2±1.0 ef	453.6±1.3 c	**487.0±2.1** **a**	390.3±0.7 h	422.1±0.7 e	458.2±0.5 c	369.3±5.0 i	420.6±0.3 ef	456.1±0.9 c	412.4±1.8 fg	449.5±0.7 c	472.2±0.7 b
N_2_	5.2±0.4 l	379.6±2.5 hi	407.5±1.2 e	442.9±2.9 bc	373.0±2.4 ij	397.5±1.7 f	442.4±0.8 bc	384.5±0.6 gh	426.7±2.2 d	**457.9±0.9** **a**	364.6±0.9 jk	391.4±1.4 fg	441.1±1.0 bc	360.4±0.9 k	385.7±1.8 gh	436.3±1.8 c	383.5±1.1 gh	417.9±1.3 d	445.8±2.9 b
N_3_	40.3±0.3 a	19.3±0.3 cd	14.7±0.3 gh	7.3±0.3 lmn	18.7±0.3 de	11.6±0.3 ij	5.7±0.3 no	17.3±0.3 def	10.4±0.3 jk	4.7±0.7 o	**23.7±0.7** **b**	16.7±0.3 efg	9.3±0.3 kl	21.3±0.3 c	15.7±0.3 fg	8.0±0.6 lm	18.0±0.6 de	13.4±0.3 hi	6.7±0.3 mno
N_4_	41.3±0.9 a	19.7±0.3 cd	16.3±0.3 ef	11.7±0.3 hi	19.3±0.3 cd	15.7±0.3 f	9.3±0.3 ij	18.3±0.3 cde	15.3±0.3 fg	8.0±0.6 j	**22.7±0.3** **b**	17.7±0.3 def	13.0±0.6 gh	20.7±0.3 bc	17.3±0.9 def	12.3±0.3 h	18.7±0.3 cde	16.3±0.3 ef	11.3±0.3 hi

Note: The data given is average ± standard deviation. Different small letters indicate a significant difference by Tukey’s new multiple range test at the P < 0.05 levels, respectively.

## Discussion

As field conditions were uncontrollable, we devised a method creating a range of variable high temperature conditions simulating field conditions in the laboratory. A range of temperatures and periods of exposure to high temperatures varied. Flies were exposed to temperatures gradually increasing from normal optimal temperature (25°C) to various highs (37°C, 45°C) of several durations (1 hr, 2 hr) before declining to normal over a 7 hr period. In this way, the response of the flies in terms of mating, oviposition and thermotaxis were studied to a range of simulated field temperature conditions. The results showed that the maximum daily temperature had an important effect on the mating, oviposition and thermotactic behaviour of the flies, and the combination of the average temperature and the highest temperature had different effects on flies mating, oviposition and thermotaxis. The maximum temperature value was 37°C with an average temperature of 33°C, which was the most conducive to mating, oviposition and thermotactic behaviour. When considering the optimal conditions for fecundity and population increase of insects, both the average temperature and the maximum temperature should be considered. All insect behaviours is influenced by changes in the environment. Flies can select favourable environment temperatures for their survival through autonomous flying and crawling. In traditional studies, flies have been passively studied under constant temperature conditions in artificial settings so they are unable to show their natural response to environmental temperatures. Research on the effects of high temperature in respect of mating, oviposition and thermotactic behaviour must take into consideration the high-temperature preferences of this fly species. The observations made in this study have practical significance for field investigations and provide a scientific basis for improving the accuracy of prediction of flies behaviour and response strategies for this and in all likelihood other insects to high temperatures. In a natural state, the mating, oviposition and thermotactic behaviours of the flies are influenced by various environmental factors in addition to temperature, including nutrition, light, humidity and other factors. The behaviour of the flies in field conditions and their relationship to temperature needs to be further studied. Exposure to a higher frequency of short-term episodes of extreme high temperatures will increase as global warming progresses. Extreme high temperatures can change microhabitat selection by an individual as well as affect the behaviour and breeding of other insects, which affects the development, breeding, and ultimately, the population development and ecosystem impact of a species. The response of insects to extreme high temperatures is species specific, and the response of different insect species to climate change also varies; for example, *Sitobion avenae* (Fabricius), *Rhopalosiphum padi* and *Schizaphis graminum* exposed to heat will exhibit crawling behaviour[6]. Extreme daytime maximum temperatures have been shown to hinder the feeding behaviour of the *L*. *coffeella*, extend the lifespan of the adult *Grapholitha molesta* (Busck), and affect the fecundity of female *B*. *dorsalis* (Hendel) and *Spodoptera exigua* (Hübner)[11,15–17]. This study showed that flies emergence, mating, oviposition and thermotactic behaviour were affected after exposure to 33°C for 1hr. Therefore, the effects of short-term extreme high temperatures on flies differ from the traditional effects of prolonged high temperatures and should be seriously considered.

Extreme high temperature is a relative concept, and current studies on the effects of extreme high temperatures on flies focus more to temperatures and heat waves that occur in summer that are near or beyond the physiological or behavioural tolerance limits of the flies. The observation and analysis of climate data show that the increase in temperature caused by global climate change is asymmetric among seasons and between the day and night. In other words, the increase in temperature in spring, autumn and winter is higher than in summer, and the increase in temperature at night is significantly lower than during the day[18]. A summer night is currently more likely to provide exposure to extreme high temperatures, but spring, autumn and winter have three times the probability of extreme high temperatures. Studies of the effects of extreme night-time temperatures on the life history and fitness of *Sitobion avenae* (Fabricius) have shown that night-time warming in the appropriate temperature range resulted in a linear decrease in the survival of the aphids[19], which differs completely from the thermostatic effects of daytime high temperatures on the performance of the adult flies in this study. Based on these findings, researchers predict that hot days and warm nights will lead to reduced populations of wheat aphids in temperate regions. When predicting the dynamics of flies under climate change, it is necessary to not only consider the strength and duration of the heat waves but also the age structure of pest populations in the field. Further study the effects of seasonal high temperatures on the development of flies in spring, autumn and winter is warranted.

## Conclusion

Results showed that 1hr of exposure to 45°C significantly stimulated mating, oviposition and thermotactic behaviour of the flies. These results could improve our understanding of the mechanisms responsible for the population dynamics of *Bactrocera cucurbitae* during periods of high temperature.
